# The Development of Plant Genome Sequencing Technology and Its Conservation and Application in Endangered Gymnosperms

**DOI:** 10.3390/plants12234006

**Published:** 2023-11-28

**Authors:** Kaiyue Hong, Yasmina Radian, Teja Manda, Haibin Xu, Yuming Luo

**Affiliations:** 1 Jiangsu Collaborative Innovation Center of Regional Modern Agriculture and Environmental Protection, Jiangsu Key Laboratory for Eco-Agricultural Biotechnology around Hongze Lake, Huaiyin Normal University, Huai’an 223300, China; awzh18252056628@163.com; 2 School of Life Sciences, Nanjing Forestry University, Nanjing 210037, China; radani.yasmina@gmail.com (Y.R.); teja.manda27@gmail.com (T.M.)

**Keywords:** genome sequencing, genetic research, biological phenomena, the conservation and utilization of gymnosperms, precision breeding

## Abstract

Genome sequencing is widely recognized as a fundamental pillar in genetic research and legal studies of biological phenomena, providing essential insights for genetic investigations and legal analyses of biological events. The field of genome sequencing has experienced significant progress due to rapid improvements in scientific and technological developments. These advancements encompass not only significant improvements in the speed and quality of sequencing but also provide an unparalleled opportunity to explore the subtle complexities of genomes, particularly in the context of rare species. Such a wide range of possibilities has successfully supported the validation of plant gene functions and the refinement of precision breeding methodologies. This expanded scope now includes a comprehensive exploration of the current state and conservation efforts of gymnosperm gene sequencing, offering invaluable insights into their genomic landscapes. This comprehensive review elucidates the trajectory of development and the diverse applications of genome sequencing. It encompasses various domains, including crop breeding, responses to abiotic stress, species evolutionary dynamics, biodiversity, and the unique challenges faced in the conservation and utilization of gymnosperms. It highlights both ongoing challenges and the unveiling of forthcoming developmental trajectories.

## 1. Introduction

Recent modern biotechnology, such as plant genome sequencing, has emerged as a pivotal tool for studying plant biology and ecology. The sequencing technique serves as plant structural organization and functional complexities and functions as a valuable resource for uncovering the plant molecular mechanisms underlying adaptive evolution, stress resilience, and agricultural characteristics [1,2].

For instance, the identification of two distinct Sichuan pepper (*Zanthoxylum armatum* and *Zanthoxylum bungeanum*) genomes has enabled the exploration of evolutionary relationships, phenotypic variations, and the interaction of adaptive evolution within the genomes of Chinese Sichuan pepper [3]. Plant genome sequencing expanded, encompassing diverse species, revealing hidden genetics influencing responses to the environment and pathogen resilience, boosting agricultural productivity [4]. Genome sequencing technology significantly impacts essential areas like genetic optimization and crop breeding advancement. A recent study had scholars conduct genome sequencing on nine wild tomato species (*Solanum lycopersicum*) and two cultivated variants, thoroughly analyzing genetic diversity and structural variations in these tomato genomes [5]. Nevertheless, the ongoing progression of technological innovation has introduced a variety of new challenges, namely in the areas of data analysis and the complex annotation of gene functionality [6]. Genome sequencing provides valuable resources for the utilization of plant resources and the conservation of endangered plants. However, the majority of genome sequencing efforts have been focused on angiosperms, making genome sequencing of gymnosperms of significant scientific and practical importance [7]. The importance of gymnosperm genome sequencing is evident in several aspects. Firstly, gymnosperms represent an ancient group of plants, and their genome research contributes to unveiling their long evolutionary history and mechanisms of adaptation to diverse environments [8]. Furthermore, gymnosperms play a significant role in ecology and ecosystem management, particularly in the stability of forest ecosystems [9,10,11,12]. Research in this field also provides the foundation for enhancing the quality and sustainable utilization of forest products, including timber, pulp, and medicinal plants [13,14]. Thus, gymnosperm genome sequencing holds profound significance for scientific research, ecosystem conservation, and economic resource development. In this review, we will delve into the developmental journey of plant genome sequencing and the latest research findings in the field of gymnosperm genome sequencing. We will explore the applications of genome sequencing technology and its implications for the conservation of gymnosperms. Through this review, we aim to stimulate greater interest in gymnosperm genome sequencing research, encouraging more scientists to engage in research in this field, and providing additional knowledge and wisdom for the development of an ecological civilization.

## 2. Advancements in Genome Sequencing Technology and Its Evolution

**Figure 1:**
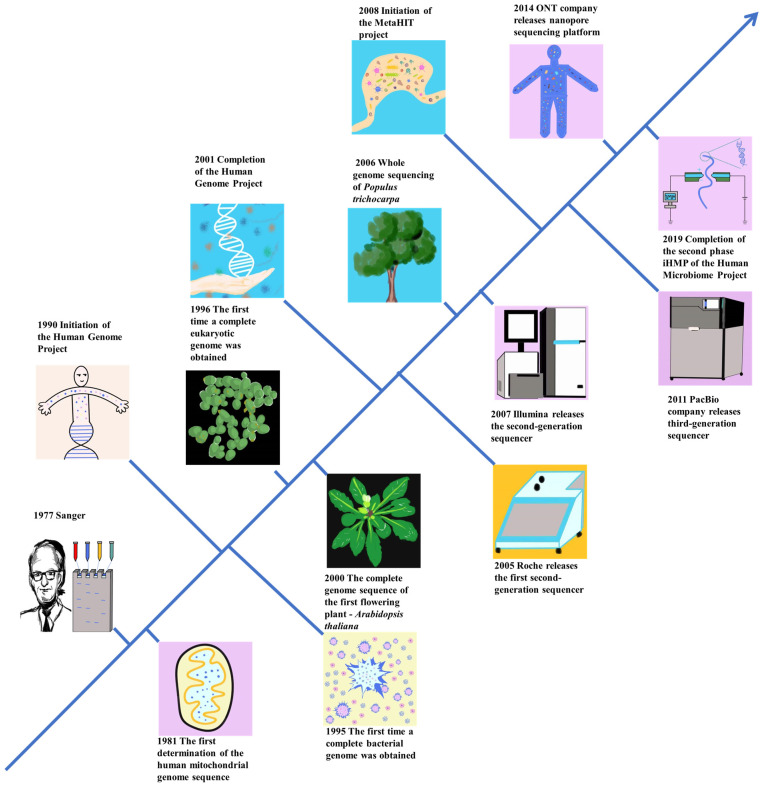
The development history of gene sequencing technology.

A faster processing speed and lower cost are responsible for the major advances in genomics. Many academics have spent the last 50 years working to create the tools and processes needed to sequence DNA and RNA molecules. This era has observed significant advancements in sequencing technology, transitioning from the analysis of individual bases to the processing of millions, and evolving from efforts to decipher the coding sequence of isolated genes to the efficient and cost-effective pursuit of whole-genome level sequencing [15]. The trajectory began with the initiation of DNA sequencing in 1977 [16], followed by the advent of Next-Generation Sequencing (NGS) in the early 2000s [17]. The early 2010s witnessed the emergence of third-generation sequencing technology, along with the progression of technological advancements [18]. The advancement of technology has not only propelled the pursuit of corresponding experimental analyses but has also catalyzed rapid progress across the entire scientific domain (Figure 1). A comparison of the advantages and disadvantages of first-generation sequencing technology, second-generation sequencing technology, and third-generation sequencing technology is shown in Table 1.

### 2.1. Pioneering Generation Genome Sequencing Technology

**Figure 2:**
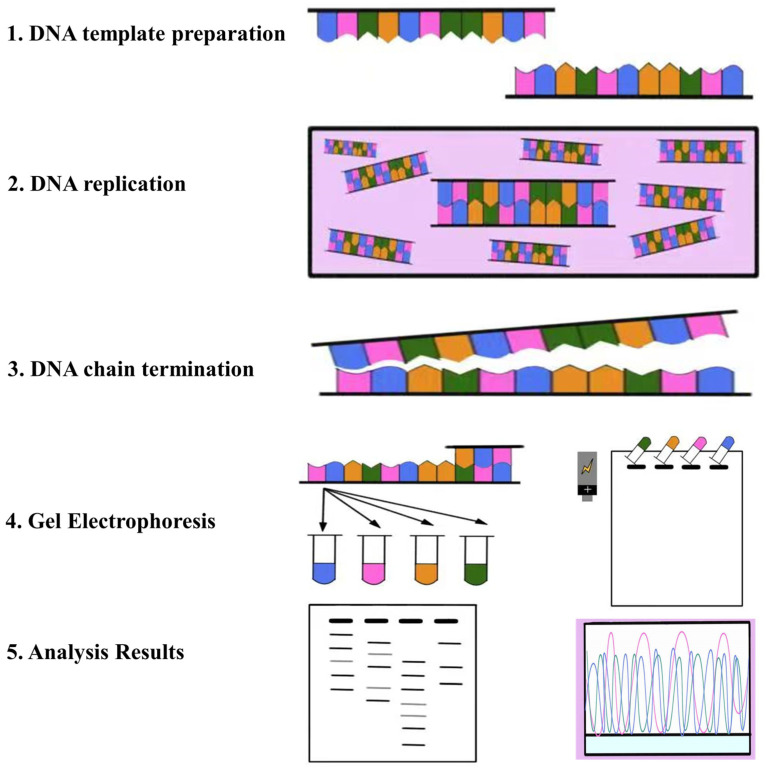
The fundamental principle of Sanger sequencing. Different colors represent different types of nucleic acids.

Sanger sequencing represents a pioneering example of first-generation genome sequencing technology [37]. It predominantly employs the dideoxynucleotide chain termination method in conjunction with the Sodium Dodecyl Sulfate Poly Acrylamide Gel Electrophoresis (SDS-PAGE). The basic idea is that dideoxyribonucleoside tri-phosphates (ddNTPs) are incorporated into the newly manufactured DNA strand and prevent the creation of phosphodiester linkages, which stops nucleotide inclusion. As a result, many DNA fragments with distinct endpoints and a similar beginning point are produced. Selectively, these pieces end at adenine (A), cytosine (C), guanine (G), or thymine (T) nucleotide bases. After the fragments are separated using polyacrylamide gel electrophoresis, the original template’s nucleotide sequence can be inferred using auto-radiography. (Figure 2).

An extended read length, modest throughput, accuracy, and dependability made Sanger sequencing the gold standard for DNA sequencing for a considerable amount of time. However, its equipment is expensive and time-consuming, which limits its widespread large-scale deployment [38].

### 2.2. Next Generation Sequencing

Genome sequencing technology is endlessly progressing in terms of cost reduction, increased throughput, and enhanced speed [39]. The development of second-generation sequencing technology followed the introduction of large-scale dideoxy sequencing, which brought to light the shortcomings of first-generation sequencing technology in satisfying the demands of high-throughput and high-quality large-scale genome sequencing. NGS is a type of high-throughput sequencing that allows for the simultaneous examination of millions of nucleic acid molecular sequences, revolutionizing sequencing methods. The next generation of genome sequencing technologies has a strong foundation thanks to this innovation. Through continuous technological refinement, second-generation sequencing technology emerged, such as Roche’s 454 technology [40], Illumina’s Solexa technology [41], and ABI’s Solid technology [42]. Unlike its predecessor, this technology does not rely on nucleotide consistency inference through pre-electrophoretic visualization using radioactive or fluorescently tagged dNTPs or oligonucleotides.

#### 2.2.1. Roche 454 Sequencing Technology

The 454 sequencing technology, also known as pyrosequencing, employs a methodology known as “sequencing by synthesis” (SBS). This technique encompasses the utilization of “water-in-oil PCR” and “pyrosequencing technology”. The current methodology involves conducting polymerase chain reactions (PCR) within confined compartments, referred to as small wells. Each cycle of the PCR reaction entails the addition of a single dNTP based on the sequence. When dNTP and sequence couple well, a pyrophosphate group is released and reacts with ATP sulfate chemase to produce adenosine triphosphate (ATP). When luciferase and freshly created ATP oxidize, light is released and recorded for sequencing using a CCD camera. The length of homomers can be difficult to measure exactly with present technology, which could result in errors and inaccuracies. Roche, who invented second-generation sequencing, shut out its 454 business in 2013 because of accuracy problems that limited platform updates and application scope [43].

#### 2.2.2. Illumina Sequencing and Solexa Technology

**Figure 3:**
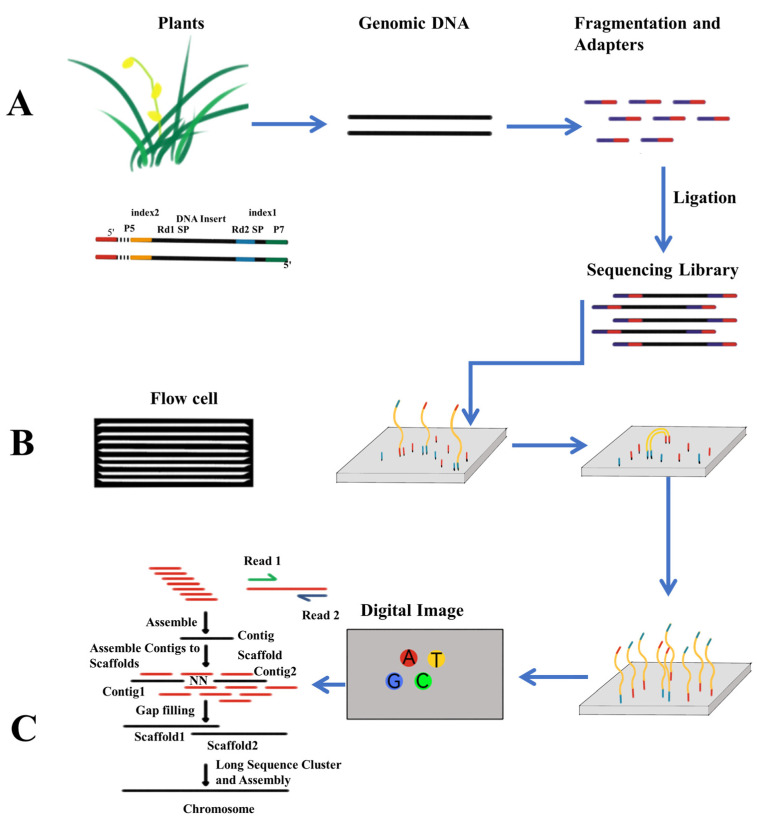
The fundamental principle of Illumina sequencing. (**A**) Library Preparation. (**B**) Cluster Amplification (**C**) Alignment and Data Analysis. Illumina P5 adapter: 5′- AATGATACGGCGACCACCGAGATCTACAC -3′. Illumina P7 adapter: 5′- CAAGCAGAAGACGGCATACGAGAT -3′. Read 1 primer: 5′- TCGTCGGCAGCGTCAGATGTGTATAAGAGACAG -3′. Read 2 primer: 5′- GTCTCGTGGGCTCGGAGATGTGTATAAGAGACAG -3′.

A multitude of parallel sequencing techniques surfaced with the introduction of 454 sequencing. Notably, the Solexa method was very significant; it was later acquired by Illumina. The “SBS” method used by Solexa sequencing entails the “reversible terminal termination reaction” and “DNA clustering” processes. The Solexa method links DNA fragments with cleaved connectors by using complementary oligonucleotides that stick to the flow pool, unlike bead-based PCR. By using arching duplicated DNA, the solid-phase PCR creates clusters from primitive flow cell-bound DNA strands, which starts the “bridge amplification” process. Solexa achieves self-sequencing utilizing fluorescent “reversible terminator” dNTPs. When the fluorophore is in the 3’ hydroxyl position, these inhibit binding. For simultaneous sequencing, the fluorophore is removed prior to polymerization. Modified dNTPs and DNA polymerases are washed cyclically in single-stranded cell-binding clusters. CCD monitors nucleotide identity via activated fluorophores, before enzymatic removal of the blocked fluorescent part, progressing to the next site (Figure 3).

Illumina released Solexa sequencing for sale in 2006. The first-generation product was the Initial Genome Analyzer (GA). Hiseq thereafter released models such as the X-ten system, Hiseq2000, Hiseq2500, Hiseq3000, and Hiseq4000. Illumina continued to grow by introducing the compact, lower throughput Miseq system and the high throughput Nextseq platform. The Miseq platform has a number of advantages, including lower costs, quicker processing, and longer read lengths [44].

#### 2.2.3. ABI SOLiD Sequencing Technology

“Sequencing while connecting” is the basic idea behind SOLiD (Sequencing by Oligonucleotide Ligation and Detection) sequencing technology. This is accomplished by employing “water-in-oil PCR” and “ligase sequencing” techniques. The library configuration is similar to the Solexa technique, which uses hybridization to attach DNA molecules to beads. Beads, PCR components, and oil are used to create separated reaction environments, which improves the sequencing template amplification process. Sequencing beads are attached to a SOLiD slide’s surface in order to facilitate the detection of sequence information. In order to decode SOLiD, a fluorescent DNA probe must connect to the target sequence in order to release signals that indicate the sequence order. The method’s 99.99% accuracy surpasses that of previous second-generation techniques. Dual reading and linked technology, which successfully addresses PCR limits in high GC regions, are used to achieve this. According to a prior study, the SOLiD sequencing tool performs better in samples with a high GC content [45].

DNA sequencer capabilities are advancing at a rate that is significantly faster than the traditional Moore’s Law trend. According to Moore’s Law, the number of transistors per unit cost, which measures the degree of complexity displayed by microchips, tends to double every two years. However, the evolution of DNA sequencing capabilities presents a remarkable contrast, with a striking doubling frequency occurring every five months from 2004 to 2015 [46]. These various fields of technological advancement exhibit differences in their functional capabilities, chemical makeup, and technical attributes. Consequently, they provide a range of unique technical platforms that researchers can customize to meet their own experimental requirements.

### 2.3. Third−Generation Genome Sequencing Technology (TGS)

**Figure 4:**
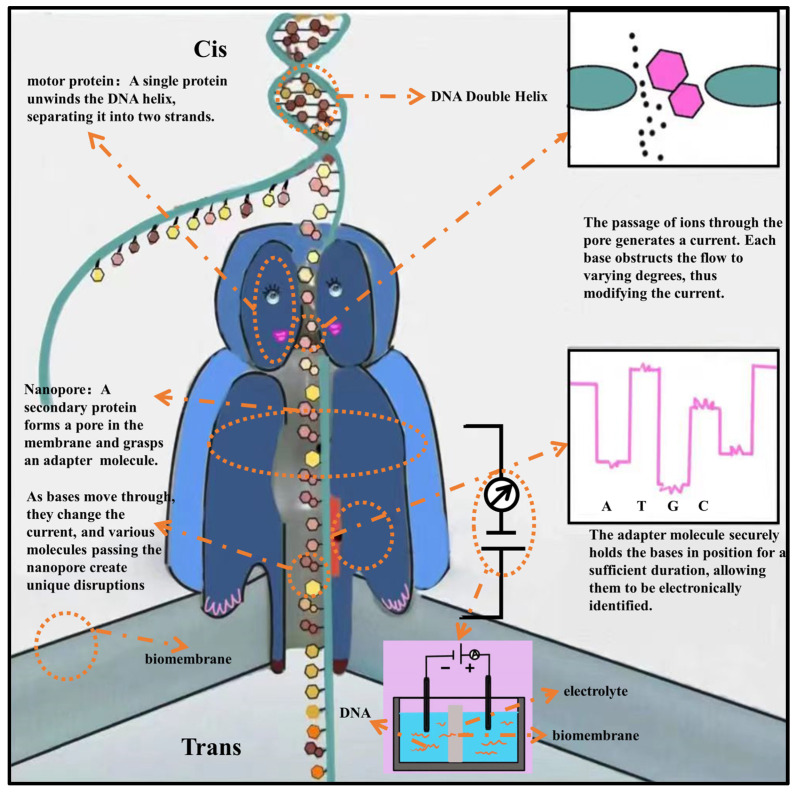
The principle of third−generation sequencing technology.

While Oxford Nanopore Technologies (ONT) and Pacific Biosciences offer long-read sequencing (more than 500 base pairs), Illumina and Thermo Fisher offer short-read sequencing platforms (between 100 and 400 base pairs). Another benefit of third-generation sequencing technologies is this [47]. The basic principles of third-generation genome sequencing technology can be seen in Figure 4.

The two leading companies in third-generation sequencing are ONT and Pacific Biosciences (PacBio). With the introduction of PacBio’s SMRT sequencing, a third-generation innovation that permits single-molecule sequencing, doing away with PCR, and permitting infinite nucleic acid sequencing, technological breakthroughs have reached a significant milestone in genome sequencing [48]. Third-generation sequencing offers several benefits, chief among them the remarkable capacity to drastically cut down on single-base mistakes, thereby circumventing earlier constraints. Because this approach is unbiased and unaffected by palindromic sequences, it can accurately identify mutations and avoid false positives. It avoids PCR, is resistant to changes caused by humans, provides long readings, guarantees even coverage, and finds methylation directly.

The SMRT technique from PacBio, which is renowned for its precision and adaptability, is revolutionizing genome sequencing. It makes use of nanostructures called Zero-Mode Waveguides (ZMWs), which have tiny holes in them to allow real-time DNA polymerization. This discovery could completely change the field of genetic research. In ZMW exposed to light, DNA polymerase elongates strands one base at a time using tagged dNTPs in real time. DNA molecule sequencing is accelerated by detectable fluorescence from recently inserted nucleotides; label removal cuts off the signal.

There are a number of benefits that set the PacBio sequencing series unique from other commercial technologies. The ability of SMRT sequencing to produce kinetic data during polymerase-driven sequencing is a crucial technical feature. This information facilitates base modification detection, which is an essential tool for de novo genome assembly [49,50]. This novel method not only speeds up the sequencing of individual molecules but also offers insightful information about genetic changes and structure. In the 1980s, the idea of nanopore sequencing first surfaced [51]. Nanopore technology analyzes alterations when biomolecules move through microscopic pores. Because of this, single-molecule sensing and analysis is possible using nanopore technology. The first iteration of ONT, called MinION, a single-molecule sequencing technology based on nanopores, was made available in 2014 [52]. Many alignments and tools for base detection, data processing, read mapping, de novo assembly, and variant discovery have now been developed by Nanopore technology [53]. Poretools is a data processing tool for MinION nanopore sequencing that supports quality control, format conversion, and data exploration [54]. Genopo is an android application for nanopore sequencing, bringing nanopore sequencing analysis to smartphones for the first time, making genetic research more convenient [55]. SquiggleKit is a toolkit for manipulating and querying nanopore data, simplifying file handling, data extraction, visualization, and signal processing [56]. A flexible toolset for analyzing DNA and RNA modifications is called ModPhred. It supports many alteration types, incorporates modification information into FASTQ and BAM files, and makes it easier to see and analyze modification data [57]. Because nanopore sequencing allows the study of genetic and epigenetic alterations and their function in gene expression easier and more accessible, it has several uses. PacBio HiFi and ONT each have their own strengths and weaknesses, as shown in Table 2.

## 3. Applications of Genome Sequencing Technology

**Figure 5:**
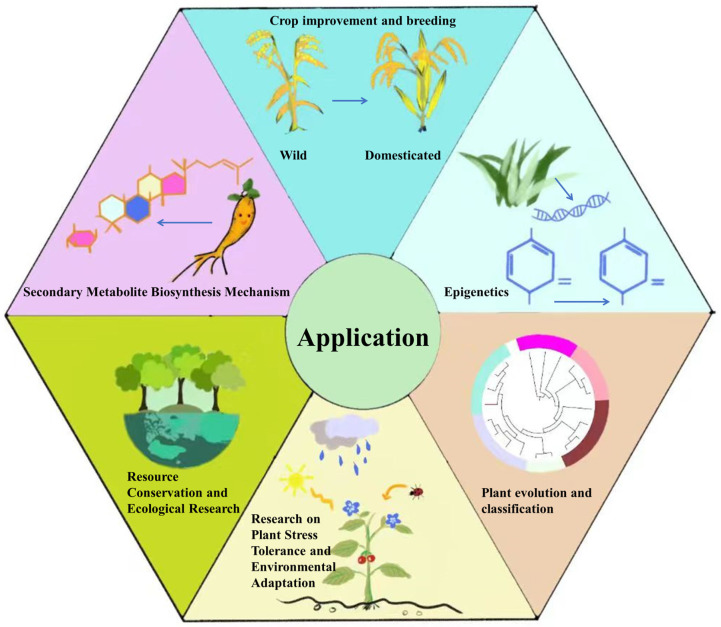
The application of genetic sequencing technology.

The PacBio RS II sequencer has been effectively utilized to generate a 1.27 Gb genome assembly of *Dendrobium officinale* [70]. By utilizing advanced sequencing technologies such as Illumina HiSeq, Nanopore, PacBio, and Hi-C, the results have revealed remarkable N50 values of 44 Mb and 65.35 Mb for *Gardenia jasminoides* and *Chimonanthus praecox*, respectively, surpassing previously perceived limitations [71,72]. Additionally, to date, it is worth noting that a substantial number of plant reference genome sequences, exceeding 800, have been officially released and are accessible to the public [73]. This surge has created new opportunities to enhance the efficiency of plant genetic research at the molecular level, enabling a deeper understanding of plant genome structure, gene composition, functionality, and the evolutionary processes of different species (Figure 5).

### 3.1. Enhancing Crop Quality through Molecular Breeding

A high-throughput sequencing technique called genotyping by sequencing (GBS) greatly broadens the pool of molecular markers that are available for crop genetics research. It is possible to use the association between these Single Nucleotide Polymorphisms (SNPs) and pertinent agronomic factors to validate trait-associated haplotypes in crops or to aid in marker-assisted breeding. Unlike other genotyping techniques, like simple sequence repeats (SSR) or restricted fragment length polymorphism (RFLP), GBS may identify a wide variety of SNPs [74]. In genotyping scenarios, including populations such as recombinant inbred lines (RILs) in rice (*Oryza sativa*), maize (*Zea mays*), barley (*Hordeum vulgare*), and double haploid (DH) populations in wheat (*Triticum aestivum*), this technology has demonstrated success [75,76,77]. Furthermore, the application of MutMap technology [78] has successfully identified the pathogenic gene OsRR22 in the rice salt-tolerant mutant hst1. The precise insights into gene transcript abundance are provided by RNA-seq-assisted expression profiling, which makes it easier to identify specific symptoms [79]. Pearl millet (*Pennisetum glaucum*), a cereal crop that is widely recognized for its exceptional heat tolerance, has been studied using a graph-based pan-genome approach in the context of genetic sequencing. This has revealed genomic variations linked to heat resilience and opened the door to cultivating more resilient crops in the face of changing climatic conditions [80]. Furthermore, the genome of the CIMBL55 maize drought-resistant germplasm resource has been assembled and annotated to a high standard thanks to the use of third-generation PacBio long-read sequencing technology, Hi-C technology, and optical mapping [81].

### 3.2. Exploration of Epigenetic Regulatory Mechanisms

Plant methylation is divided into two types: Cytosine methylation (C-methylation) and Adenine methylation (A-methylation) [82]. Joint-snhmC-seq is a novel technique for accurately determining 5-methylcytosine (5mC) and 5-hydroxylmethylcytosine (5hmC) levels in DNA at the single-cell level, offering advantages of low sample input, high accuracy, and simultaneous detection of both modifications [83]. Recently developed software, such as DeepSignal-plant (version 1.2.0), combines deep learning and nanopore sequencing to detect methylation information in plant genomes [84]. Analysis of Methylation-Sensitive Amplified Fragment Length Polymorphism (MS-AFLP or MSAP) is commonly used to assess changes in response to methylated cytosine under various stimuli and has recently been applied in ecological studies of wild plant populations [85].

A-methylation is a common modification in RNA, including N6-methyladenosine (m^6^A) and N1-methyladenosine (m^1^A) [86]. Among these two, m^6^A is considered the most common, abundant, and evolutionarily conserved internal transcription modification in eukaryotic mRNA [87]. Nanopore sequencing is a method that does not require heavy sulfuric acid conversion or immunoprecipitation enrichment experiments. It enables the direct simultaneous detection of C5-methylcytosine and m6A during sequencing [88]. In recent times, the utilization of the ONT platform for direct RNA sequencing (DRS) has been considered a promising alternative method for studying m^6^A [89].

In Phyllostachys edulis, the application of DRS for the modification of circular RNA has successfully achieved the precise detection of m^6^A, revealing the presence of m^6^A modifications in circular RNA and their distribution within exonic circular RNA [90]. Through the use of an improved rice genome and SMRT sequencing technology, researchers have successfully identified genome-wide m^6^A sites in both indica and japonica rice genomes at a single-nucleotide resolution. Concurrently, they observed a positive correlation between m^6^A and the expression of key genes associated with heat stress. Subsequently, they conducted further screening for potential mutants related to epigenetics [91]. In petunia petals, a comprehensive transcriptome analysis of RNA containing an m1A modification was conducted by combining LC-MS/MS, dot blotting, and methylated RNA immunoprecipitation sequencing (MeRIP-seq or m^1^A-seq). The study revealed that ethylene treatment decreased the overall mRNA m1A peak in the petals [92]. These studies have provided valuable information for a deeper understanding of the mechanistic roles of m^6^A and m1A in different biological systems.

Techniques for detecting DNA methylation fall into three categories. Whole-genome investigations utilizing a variety of methods, including nanopore sequencing, whole-genome bisulfite sequencing (WGBS), DNA methylation immunoprecipitation (MeDIP), and reduced representation bisulfite (RRBS) [93]. Advances in sequencing technologies have led to a significant expansion in the field of studying plant epigenetics in a variety of contexts. Significantly enhancing our understanding of how plants epigenetically respond to environmental signals has, in turn, facilitated plant adaptation.

Bisulfite pyrosequencing is a quick gold standard for methylation, and NGS, like WGBS, facilitates comprehensive genome-wide analysis of DNA methylation changes [94]. WGBS, a robust molecular experimental technique, enables the precise examination of methylation status at individual CpG sites with high resolution [95]. WGBS treats DNA with sodium bisulfite, converting unmethylated cytosines to uracils while preserving methylated cytosines. High-throughput sequencing compares results with the reference genome to identify methylated sites and levels [96]. In *Morus alba*, the WGBS methylation analysis unveiled genomic modifications in response to drought stress, thereby enhancing our comprehension of the intricate relationship between DNA methylation and the regulation of gene expression under abiotic stress conditions [97]. Similarly, the application of analytical methods such as WGBS revealed a correlation between tanshinone accumulation and the methylation levels of key enzyme genes, underscoring the significance of CHH methylation in the regulation of tanshinone biosynthesis [98]. In *Fragaria vesca*, WGBS studies brought to light that FDM1 regulates gene expression through CHH methylation, particularly at the promoter and 3’ end, exerting an impact on DNA methylation levels and influencing both plant height and fruit size [99]. In *Glycine max*, the utilization of WGBS demonstrated the heritability of DNA methylation variation [100]. However, due to short read lengths, WGBS is unable to analyze repetitive genomic regions or regions with 5mC in PCR-biased areas.

MeDIP is an additional accurate technique for analyzing DNA methylation. Methylation-sensitive restriction enzyme sequencing (MRE-seq) can be combined with MeDIP to improve the precision of methylation investigations [101]. The combination of MeDIP and MRE-seq serves to further refine the precision of methylation studies [102]. In *Prunus avium*, DNA methylation levels were analyzed through MeDIP, revealing that *PavMADS1* and *PavMADS2* are intriguing candidate genes involved in regulating flowering. Additionally, they play a crucial role in regulating dormancy in sweet cherries [103]. Global MeDIP-Seq in young and aging *Gossypium hirsutum* revealed lower DNA methylation in aging cotton leaves. Reduced DNA methyltransferase activity is key to regulating secondary metabolites [104].

RRBS utilizes a methylation-sensitive restriction endonuclease to cleave unmethylated DNA into fragments enriched in high GC-density CpG sites. Following additional processing and selection steps, these fragments undergo bisulfite conversion, PCR amplification, and sequencing [96]. RRBS is extendable for ecological experimental designs, applicable to organisms without a reference genome, and exhibits higher resolution compared to previous marker-based methods [105]. Platt et al. employed RRBS in *Quercus lobata*, revealing stronger population differentiation at SMPs than SNPs, suggesting epigenetic heritability [106]. RRBS methylation quantification has been widely employed in large-scale sample analyses of plant methylation profiles, providing evidence for Epigenome-Wide Association Studies (EWAS) [107]. Schmitz et al. conducted RRBS research on 83 soybean Recombinant Inbred Lines (RILs) and their parents, aiming to identify patterns of methylation variation and heritability. The study sought to gain a deeper understanding of how methylation variation contributes to phenotypic diversity [108].

Studying the methylation of mammalian cells helps regulate genes, better understand diseases, and direct the creation of treatments. In order to ensure genomic integrity, silence genes, and promote development, DNA methylation is essential [109]. The analysis of 15,000 samples from 348 mammal species revealed a close link between DNA methylation patterns and genetic evolution [110]. Longer-lived species exhibit distinct methylation peaks and valleys in their genomes [111]. Furthermore, researchers can learn more about the function of methylation in gene regulation by comparing methylation patterns under various physiological or pathological conditions. DNA methylation in mammals may be one of several elements preventing cancer in large, long-lived species [112]. For example, by suppressing repetitive DNA elements that threaten genome integrity or by limiting the developmental plasticity of differentiated cells [113].

### 3.3. Evolutionary Analysis of the Origin of Species

Through the lens of comparative genomics, the emergence of new genomic resources made possible by the development of genome sequencing technology has not only improved our comprehension of the developmental processes in land plants but also revealed the ancient evolutionary origins of plants. Using genome evolution analysis and transcriptome sequencing in a phylogenetic context, some fascinating discoveries have been made. Recent findings have shown that among the existing land plants (embryonic plants), there are a large number of novel species of vascular plants that are all related to a common ancestor [114].

Notably, a comparative analysis of the genomes of deciduous and evergreen trees has produced some interesting results. Interestingly, Siberian Larch (*Larix sibirica*) has been found to harbor prominent expression of the genes regulating EXL2 and DRM1 proteins, while evergreen trees have been found to harbor an overabundance of genes regulating immune receptor proteins [115]. The advancement of sequencing technology has not only produced essential references for interpreting the genomes of *Hordeum vulgare* and *Triticum aestivum*, but it has also established a key framework for addressing outstanding questions regarding the genomics of the domestication of wheat and barley [116]. The genome of a polyploid cultivar of *Chrysanthemum morifolium* was successfully deciphered in a recent study, which provides the first report of a segmental allo-polyploid genome worldwide and provides extensive insight into the history of breeding and origin of cultivated *Chrysanthemum morifolium* [117]. The genome of *Chimonanthus praecox* offers insights into the molecular mechanisms that drive magnolia evolution and petal color development [118]. Moreover, by the utilization of large-scale genetic data and complex data analysis techniques, the research has fully resolved the mysteries surrounding the genesis, domestication, and migration of grapes. By addressing many debates within the scholarly grape community, this research has created a cohesive viewpoint on the origin and migration of grapes. Consequently, the current storyline found in grape research textbooks has been updated [119]. Recent research initiatives have drawn special attention to the in-depth historical account of the careful human selection and nurturing of individual wheat grains in a variety of environmental conditions. This thorough investigation has brought to light its importance as a valuable collection of genetic variants and as a crucial tool in the field of wheat breeding techniques [120].

### 3.4. Biodiversity

The first reference genome assembly for the high-extinction-risk Qinling serow has been made possible in large part by the use of HiFi sequencing technology in the field of animal conservation. This historic achievement has since laid the groundwork for investigating the evolutionary history of the Qin-ling serow and clarifying the fundamental causes of its elevated extinction risk [121]. Technological innovation resulting from the genetic revolution has profound consequences for the conservation of plant resources and their inherent diversity, as well as for the protection of animal resources [122]. Noteworthy research has underscored the capacity of individual plant genes to exert influence over the species diversity within entire ecosystems [123]. Beyond its impact on the diversity of other species, plant sequencing carries profound ramifications for the internal diversity of plant species themselves, as well as their conservation endeavors.

Genome sequencing is becoming a powerful method for identifying genetic variety in plant populations. One such instance is the correlation between comprehensive whole-genome SNP data and phenotypic information obtained from ginkgo seed nuclei. In order to preserve and utilize the domesticated germplasm of this living fossil plant, this integration has effectively identified correlations between 54 SNPs and a variety of ginkgo properties [124]. Furthermore, an insightful theory regarding the development of plastid genome diversity, which is influenced by intricate interactions with the nuclear genome has been put forth by a study that harmonizes Illumina and PacBio sequencing datasets [125].

Prominent accomplishments in this field include the painstaking assembly of the hawthorn genome, which has yielded new understandings of the dynamics of the hawthorn plant variety and its evolutionary adaptation. As such, this accomplishment serves as a crucial point of reference for future post-genomic investigations in the hawthorn genus [126]. Researchers who have used genotype-by-sequencing (GBS) technology have examined the germplasm of four different *Brassica oleracea* subspecies in detail. This meticulous project has shown that the allelic diversity of the original broccoli species is higher than that of its hybrid equivalents by a ratio of 4.8, highlighting the possibility of maintaining this variety to improve the quality of broccoli [127]. Notably, the fields of genomics and species conservation have been effectively linked by the invention of seedeR, a predictive tool that makes use of genetic offsets and allele frequency transformation functions. This instrument can predict the genetic similarity between known sources of germplasm and particular target locus [128].

Furthermore, the application of genome sequencing has become a powerful tool for protecting threatened plant species. This species is in danger of going extinct because of adaptive gene degradation linked to stress responses and habitat expansion, which has been brought about by the *Circaeasteraceae Ranunculales*’ dependence on stress-free habitats, as revealed by the genetic analysis [129]. A thorough re-sequencing analysis has revealed that climatic change plays a critical role in Davidia involucrata’s susceptibility. As such, climate sensitivity is one of the most important factors that must be carefully taken into account in the effort to protect this species [130]. The critically endangered Rhododendron griersonianum genome was sequenced using PacBio and Illumina technologies, along with Hi-C-assisted genome assembly and population genetics analysis. This case study serves as an example of how important it is for conservation initiatives to limit opportunities for inbreeding in order to ensure the maintenance and perpetuation of the population [131]. Ostrya rehderana’s re-sequencing has provided complementary findings that highlight the urgent need to prevent inbreeding from causing sharp declines in population genetic diversity, as this constitutes a concealed threat to the species’ capacity to adapt to shifting environmental conditions [132].

### 3.5. Abiotic Stress and Biotic Stress

*Pennisetum glaucum*’s pangenome construction has produced important genetic resource information for future study in related domains. Furthermore, a thorough comprehension of the molecular processes underlying this species’ ability to withstand heat has been attained, providing insight into the role that structural variations play in heat stress reactions [80]. A total of 102 genotypes of *Zea mays* were resequenced under both control and heat stress conditions. The results showed alterations in gene expression and genomic regulatory regions associated with responses to heat stress [133]. Furthermore, the examination of reference genome sequences from both farmed and wild varieties of *Phaseolus vulgaris* revealed insights into the mechanisms underlying their ability to recover from moderate heat stress and the decrease in the reservoir of genes associated with disease resistance [134].

#### 3.5.1. Genes/QTL and Plant Stress

One or two key genes/QTL that give high resistance often govern resistance. For plants to respond to biotic and abiotic challenges, gene/quantitative trait locus (QTL) must be identified, localized, and stacked [135]. A basis for comprehending significant phenotypic and genetic linkages connected to early-stage drought resistance in *Triticum aestivum* was established by GWAS and BPP QTL co-mapping [136]. Rice resilience is increased by the enhanced Tapaswini rice variety, which has six gene/QTL for resistance to both biotic and abiotic stressors and four BB-resistant genes stacked [137], utilizing conventional gene mapping techniques to find and locate disease-resistance genes in order to create high-yielding, stress-tolerant *Pisum sativum* cultivars [138]. Creating molecular genetic markers and applying these markers to QTL analysis is a method that is being used more and more frequently in crop breeding programs to enable complicated quantitative trait selection [139]. It is now feasible to quickly and effectively create DNA markers, fine map, and identify candidate genes for biotic resistance in mung beans thanks to the reference genome sequence of the bean and modern advanced sequencing technologies [140].

#### 3.5.2. DNA Methylation and Plant Stress

Plants exhibit three distinct forms of methylation, namely mCG, mCHG, and mCHH [119]. While WGBS is not appropriate for repeating sequences, it is mostly used to detect cytosine methylation. Methylation detection may be impacted by bisulfite treatment and DNA degradation. Sequential methylation can be directly detected by Nanopore and Pacbio SMRT sequencing, which works well for repetitive sequences [84].

DNA methylation is a highly significant epigenetic modification [141]. Unexpectedly, DNA methylation controls the expression of some plant defense genes in response to biotic stress. Distinctive differential methylation patterns can be triggered by various stress circumstances [142]. DNA methylation influences chromatin accessibility and histone modifications, which in turn controls the expression of nearby and distant genes throughout the domestication process of rice. Domesticated rice experiences a decrease in the DNA methylation linked to stress tolerance, but weedy rice, which can withstand more severe stress, may show an increase in this link [143]. The drought response regulatory network of *Fragaria nilgerrensis* was revealed by means of a thorough examination of gene expression profiles, whole-genome DNA methylation maps, and physiological parameters at four distinct time points under drought stress treatment [144]. The methylation-sensitive amplified polymorphisms (MSAP) technique is a key technology for studying the dynamic changes in DNA methylation [145]. A study that looked at how cold stress affected the DNA methylation in maize seedlings discovered that a quick and beneficial epigenetic response of the plants to the stress is the demethylation of particular genes, which advances our knowledge of their adaptive mechanisms [146]. In gymnosperms, high levels of methylation enable the cycad to maintain stability and integrity throughout its evolutionary process [147]. DNA methylation studies show that genes linked to seed growth, such as those involved in lipid and cell wall synthesis, are found in demethylated regions of ginkgo seed genomes. These modifications may also have an impact on the production of energy [8]. In the genome of *Pinus tabuliformis*, there are numerous repetitive sequences, and significantly expanded gene families are primarily associated with stress responses [148]. A significant portion of these sequences are transposons that come from archaic viruses and can be harmful to the genome. Chinese pine therefore depends on high methylation levels to inhibit their function [149]. In ginkgo, differential expression of DNA methylation-related genes may be related to the gender determination of ginkgo leaves [150]. In *Pinus radiata*, high-temperature stress results in reduced methylation in embryogenic callus and somatic plants [151].

### 3.6. The Synthesis of Secondary Metabolites in Plants

An increasing number of plant genomes and pan-genomes have been thoroughly characterized thanks to the continuous progress in sequencing technology. Comprehensive datasets like these are important resources for revealing the genetic foundations of metabolic variation [152]. Utilizing *Pueraria lobata* as a case study, researchers employed PacBio and Hi-C sequencing methodologies to attain a superior genome assembly, thereby revealing its complex attributes. The biosynthesis pathways of essential secondary metabolites have been studied using multi-omics techniques, providing insights into resource efficiency and the possibility of improving genetics through breeding [153]. Researchers have successfully used multi-omics techniques to shed light on the intricate biosynthesis of terpenoids and flavonoids found in the dimorphic floral structures of the biennial *Sinoswertia tetraptera* that thrives in high-altitude domains [154]. ONT and Illumina sequencing. along with sophisticated assembly techniques, were used in the *Catharanthus roseus* investigation. This all-inclusive method investigated chromatin interactions within a well-assembled genomic structure using Hi-C data. The results showed how different metabolite synthesis patterns are guided by chromosomal conformation, which controls the expression of genes specific to organs [155].

### 3.7. Conservation of Endangered Gymnosperms

#### 3.7.1. Current Status of Gene Sequencing in Gymnosperms

Only in the last ten years have entire genome assemblies for gymnosperms been accomplished, owing to their remarkably enormous genome sizes [7]. Early in 2013, the *Pinus taeda* assembly was first suggested, representing the first gymnosperm species genome draft [156]. With the advancement of technology, an increasing number of gymnosperm whole genomes have been sequenced. Below is a list of currently available gymnosperm whole genome assemblies (Table 3).

Gymnosperms often show paternal inheritance for chloroplasts (pollen flow and trace gene flow direction) and maternal inheritance for mitochondria (seed flow and population migration history). Currently, Illumina sequencing is used to sequence the genomes of both chloroplasts and mitochondria in the majority of gymnosperms [173]. The chloroplast genome provides insights into the exploration of the evolution and relationships among gymnosperms. The recent phylogenetic relationship between *Tsuga longibracteata* and *Tsuga chinensis* is of particular interest [174]. An increasing number of gymnosperms have been the subject of chloroplast genome research, such as *Cycas Szechuanensis* [175], *Cycas ferruginea* [176], *Ginkgo biloba* [177], and *Podocarpus imbricatus* [178]. Large repeat sequences play a crucial role in the evolution and recombination of chloroplast genomes in gymnosperms, as confirmed in chloroplast genome studies of *Picea schrenkiana* [179], *Picea rubens* [180], and *Pinus wangii* [181]. *Cycas hongheensis* is ranked as an extremely endangered species by the International Union for Conservation of Nature (IUCN) Red List of Endangered Species. The chloroplast genome will facilitate the investigation of the phylogeography of Cycadaceae plants and enable more comparative research of chloroplast genomes within the Cycadaceae family [182]. Combining PacBio long-read sequencing data with Illumina short-read sequencing data from a single haploid megagametophyte allowed for the assembly of the first mitochondrial genome of *Abies alba*. Analysis of the mitochondrial genome sequencing showed significant structural and compositional heterogeneity [183].

#### 3.7.2. Gene Sequencing Technology and Endangered Gymnosperms

The national key protected wild plant list, as of 2021, includes seven gymnosperm families: Cycadaceae, Ginkgoaceae, Podo-carpaceae, Cupressaceae, Taxaceae, Pinaceae, and Ephedraceae. Based on the examination of DNA sequences and information from 115 microsatellite locus (SSR), certain genetic characteristics have been identified in Cycas simplicipinna, an endangered species in the Cycadaceae family. These include a marked genetic structure, a notable level of genetic variation among groupings, and recent population decreases. These findings can provide guidance for the conservation of this endangered species in order to prevent its extinction [162]. After resequencing 545 ginkgo genomes worldwide, three refugia and the ancient genetic components of ginkgo were discovered. This has made it feasible to draw inferences about the genetic composition and interactions amongst ginkgo populations, providing invaluable genomic resources for addressing a variety of issues related to living fossil species [163]. Genomic research has uncovered the strategies used by ginkgo biloba to expand its genome and has also identified important genes involved in the formation of the seed flagellum. With the exception of ginkgo and horsetail plants, these genes have vanished from all seed plants, providing insight into the evolution of gymnosperms [146]. *Pherosphaera hookeriana*, a plant of the Podocarpaceae family, is endangered. Utilizing SSR markers as a tool to assess genetic diversity and organization, the findings revealed evidence of a post-glacial genetic bottleneck in *P. hookeriana*. Nonetheless, some of its diversity has survived, thanks, in part, to the maintenance of gender differences, geographic range, and population continuity after the ice age. One of the biggest challenges to the survival of this species is still preventing fires [164]. In order to better understand *Cupressus chengiana*, 884.82 high-quality SNPs from 266 different *Cupressus chengiana* samples were identified, identifying this endangered species in the Cupressaceae family. Analyzing the population genetics of the species across its whole range was the aim of this assessment. In population genomics, high-throughput sequencing (HTS) can reconstruct the complex evolutionary history of mountainous species that are endangered, providing crucial data for conservation initiatives and advancing our comprehension of the enormous biodiversity found in mountainous regions [184].

Genetic diversity among wild populations of *Taxus wallichiana* was measured using Random Amplified Polymorphic DNA (RAPD) and Amplified Fragment Length Polymorphism (AFLP). The study’s findings demonstrated a rough correlation between population distribution patterns and genetic differentiation. The optimal conservation strategy for *T. wallichiana* is to safeguard all populations off-site while also safeguarding select populations on-site. APD and AFLP, or amplified fragment length polymorphism and random amplified polymorphic DNA, were employed to quantify genetic variation in *Taxus wallichiana* wild populations [166]. The study’s findings demonstrated a rough correlation between population distribution patterns and genetic differentiation. The optimum conservation strategy for *T. wallichiana* is to protect all populations off-site while also safeguarding certain populations on-site [162]. Using Direct Amplification of Mini-Satellite DNA (DAMD) and Inter-Simple Sequence Repeat (ISSR) markers, the genetic variability and population structure of Ephedra foliate were evaluated [185]. The results of the study imply that the high levels of genetic differentiation and moderate gene flow seen in the species may be caused by elements such as geographic isolation, regional climate conditions, overexploitation, and incorrect seed harvesting [186].

## 4. Discussion

First-generation sequencing techniques are no longer as useful in modern genome sequencing projects; they are now mostly useful for smaller-scale sequencing jobs like point mutation detection and clone verification [187]. Especially in more specialized applications, conventional methods such as Sanger sequencing, TA cloning, and online tools for DNA methylation detection have been used [188].

The rising use of second-generation sequencing can be ascribed to its benefits, which include high throughput and affordability. The read lengths of second-generation sequencing are often shorter, varying between 35 and 700 base pairs. Along with Insertions/Deletions (InDels), these short-read sequencing methods have been essential in advancing innovative reference genome assembly, deciphering the complexities of population structure, and identifying SNPs [189,190].

Third-generation sequencing systems, on the other hand, such as PacBio and Oxford Nanopore, provide long-read sequencing technologies that can reach thousands of base pairs, surpassing the limitations of short-read sequencing and providing researchers with creative alternatives. By avoiding the biases associated with reverse transcription and amplification, long-read sequencing technology allows for the direct sequencing of individual molecules, opening up new avenues for functional genomics research in the field of plant biology [48].

Through the combination of Hi-C and Third-Generation Sequencing (TGS), recent studies have successfully assembled Telomere-to-Telomere (T2T) genomes for a number of plant species [191]. The research team led by Dr. Su Xiaohua used a hybrid strategy that combined second and third-generation sequencing technologies to achieve a reliable chromosome-level genome assembly for *Populus Koreana* [192].

The selection of different sequencing technologies holds the potential for beneficial outcomes across multiple fields of study. A software tool has been developed for designing Small Guide RNAs (sgRNAs) applicable to all sequenced species [193]. Additionally, a novel plant gene functional annotation software, GFAP (3.8 version), has been introduced, demonstrating the ability to efficiently annotate more than 2000 genes with GO, KEGG, and Pfam information in just 4.5 s. This efficiency surpasses the performance of current mainstream functional annotation tools [194]. The future is expected to witness the emergence of additional software solutions tailored to gene sequencing, facilitating enhanced research endeavors for scholars in the field.

Plant genome sequencing still has certain challenges, especially when it comes to creating comprehensive, intricate, and pan-genomes for plants. Large genome sizes, heterozygosity, and polyploidy are only a few of the factors that continue to be very difficult. Plant genome decoding is a challenging task because of the enormous variations in genome size [195]. For instance, the genome of the lily family’s Fritillaria exceeds 100 Gb, despite spiral algae (Spirogyra) having only 63 Mb. This discrepancy in size is partly explained by the presence of large amounts of non-coding DNA sequences [6].

While cross-species comparative genomics is becoming more and more popular, access to many population genomics datasets is limited, which hinders additional research. In order to encourage data sharing, researchers in the field of population genomics are calling for a more transparent and cooperative approach [75]. Furthermore, the discourse presently encompasses perspectives on the preservation endeavors and utilization of gene sequencing in gymno-sperms, tackling their distinct obstacles and inputs to the wider domain [73].

## 5. Conclusions

To summarize, the increasing adoption of genome sequencing technology has emerged as a crucial pillar of modern plant science. The ongoing development of sequencing methods portends a time when plant genome sequencing precision will rise to previously unheard-of levels, providing dramatic improvements in the reliability and quality of data. This trajectory has the potential to offer more precise and all-encompassing support to researchers, enabling the investigation of the complex structure, functional dynamics, and composition of plant genomes.

Advances in technology are shedding light on mysterious features found in plant genomes and providing a greater knowledge of genetic information. These significant realizations provide a strong scientific basis for tackling pressing issues in ecology, agriculture, and environmental preservation. With the advancement of genome sequencing technology, important aspects such as stress tolerance, plant adaptability, evolutionary processes, and the complex interactions of genetic diversity will soon be explored. Consequently, this creates a strong basis for upcoming sustainable development projects.

Throughout this evolutionary process, a clear pattern appears that points to the widespread use and ongoing improvement of plant genome sequencing. The possibility of accurately modifying plant genomes is enhanced by the continuous development of various gene editing and synthetic biology technologies, opening up new avenues for breeding and development. Concurrently, techniques controlling the analysis and interpretation of genome sequencing data are constantly improved and optimized, guaranteeing the precise and significant insights that are extracted from large datasets.

Recognizing the unique contributions made by genome sequencing technology to the preservation and use of genetic data in gymnosperms is crucial in this regard. The use of gene sequencing methods in gymnosperm conservation raises the bar for the profession and emphasizes how crucial it is to comprehend and maintain the genetic diversity present in these ancient plant species. To summarize, genome sequencing technology is going to have a significant and long-lasting influence on the direction of plant science, offering solid and essential backing for investigating mysterious features of the plant kingdom. With technology developing at a breakneck pace, our understanding of plant genomes will inevitably grow, providing a solid scientific foundation that will enable humanity to map out a brighter, more enlightened future.

## Figures and Tables

**Table 1:** Comparison of the Advantages and Disadvantages of First-generation Sequencing Technology, Second-generation Sequencing Technology, and Third-generation Sequencing Technology.

Name	Advantages	Disadvantages	Reference
Pioneering Generation Genome Sequencing Technology (Sanger)	Offers reliable data for small-scale projects.	Slower, for small-scale projects.	[19,20]
	Fits shorter DNA sequencing like gene or Sanger sequencing.	Costly reagents and equipment expenses.	[21,22]
	Mature workflows and analysis tools after years of development.	Can’t meet high-throughput sequencing needs.	[23,24]
NGS	Fast sequencing of many DNA fragments for high-throughput projects.	Produces shorter reads, limiting applications like complex genome assembly.	[25,26]
	Relatively inexpensive for large-scale sequencing.	Generates abundant data, and needs intricate processing and analysis.	[27,28]
	Suitable for multi-sample sequencing for population and ecology studies.	Some techniques introduce sequencing biases or errors.	[29,30]
Third-generation genome sequencing technology (TGS)	Long-read sequencing aids complex genome assembly and detection.	Longer data generation time is not ideal for high-throughput sequencing.	[30,31]
	No PCR, fewer errors.	Demands more computing and complex data analysis.	[32,33]
	Useful for genome structure research: Reveals repetitive sequences and chromosomal rearrangements with long reads.	High cost.	[34,35]
	Provides real-time data for monitoring DNA synthesis and modifications.		[36]

**Table 2:** Comparison of the Advantages and Disadvantages of PacBio HiFi and ONT.

Name	Advantages	Disadvantages	Reference
PacBio HiFi	Long reads aid in complex genome assembly and detection.	Lower throughput extends data generation time.	[58]
	High accuracy lowers errors.	High cost.	[48,59]
	Direct DNA sequencing, no PCR, fewer errors.		[60]
	Useful for genome structure research: Reveals repetitive sequences and chromosomal rearrangements with long reads.		[61]
Nanopore ONT	Offers real-time data for monitoring DNA synthesis and modifications.	Low accuracy, requires multiple runs for data quality.	[62,63]
	Compact and lightweight device.	Complex data processing needs more computing resources and tools.	[64,65]
	Enables real-time data analysis, accelerating the research process.		[66,67]
	Lower sequencing costs.	Relatively shorter read lengths are unsuitable for some long-read studies.	[68,69]

**Table 3:** List of currently available gymnosperm whole-genome assemblies.

Name	Methods	Size	Reference
*Pinus taeda*	Sanger + Illumina	23 G	[156]
*Picea glauca*	Illumina	23.6 G	[157]
*Pinus lambertiana*	Illumina	27.6 G	[158]
*Pseudotsuga menziesii*	Illumina	15.7 G	[159]
*Abies alba*	Illumina	18.2 G	[160]
*Larix sibirica*	Illumina	12.3 G	[161]
*Taxus wallichiana*	Illumina	10.9 Gb	[162]
*Taxus chinensis*	PacBio + Hi-C + Illumina	10.23 Gb	[163]
*Taxus yunnanensis*	Illumina + Nanopore	10.7 Gb	[164]
*Sequoiadendron giganteum*	Illumina +Hi-C + Nanopore	8 GB	[165]
*Picea abies*	Shortgun	20 GB	[166]
*Pinus tabuliformis*	PacBio + Hi-C + Illumina	25.4 Gb	[149]
*Larix kaempferi*	PacBio + Bionano + Illumina	10.97 GB	[167]
*Ginkgo biloba*	PacBio + Hi-C	9.88 Gb	[168]
*Gnetum montanum*	Illumina	4.5 G	[169]
*Welwitschia mirabilis*	Illumina + Nanopore	6.86 Gb	[147]
*Cycas panzhihuaensis*	PacBio + Illumina	10.5 Gb	[170]
*Torreya grandis*	PacBio + HiFi + Illumina	19 Gb	[8]
*Metasequoia glyptostroboides*	ONT + Illumina + Hi-C	8.07 Gb	[171]
*Sequoia sempervirens*	PacBio HiFi	27 Gb	[172]

## Data Availability

This article is a review and does not contain original data.

## References

[1] Marks R.A., Hotaling S., Frandsen P.B., VanBuren R. (2021). Representation and participation across 20 years of plant genome sequencing. Nat. Plants.

[2] Zhou Y., Li K., Wen S., Yang D., Gao J., Wang Z., Zhu P., Bie Z., Cheng J. (2023). Phloem unloading in cultivated melon fruits follows an apoplasmic pathway during enlargement and ripening. Hortic. Res..

[3] Hu L., Xu Z., Fan R., Wang G., Wang F., Qin X., Yan L., Ji X., Meng M., Sim S. (2023). The complex genome and adaptive evolution of polyploid Chinese pepper (*Zanthoxylum armatum* and *Zanthoxylum bungeanum*). Plant Biotechnol. J..

[4] Peng Y., Jin M., Li Z., Li H., Zhang L., Yu S., Zhang Z., Fan R., Liu J., Xu Q. (2023). Population Genomics Provide Insights into the Evolution and Adaptation of the Asia Corn Borer. Mol. Biol. Evol..

[5] Li N., He Q., Wang J., Wang B., Zhao J., Huang S., Yang T., Tang Y., Yang S., Aisimutuola P. (2023). Super-pangenome analyses highlight genomic diversity and structural variation across wild and cultivated tomato species. Nat. Genet..

[6] Kress W.J., Soltis D.E., Kersey P.J., Wegrzyn J.L., Leebens-Mack J.H., Gostel M.R., Liu X., Soltis P.S. (2022). Green plant genomes: What we know in an era of rapidly expanding opportunities. Proc. Natl. Acad. Sci. USA.

[7] Wan T., Gong Y., Liu Z., Zhou Y., Dai C., Wang Q. (2022). Evolution of complex genome architecture in gymnosperms. GigaScience.

[8] Lou H., Song L., Li X., Zi H., Chen W., Gao Y., Zheng S., Fei Z., Sun X., Wu J. (2023). The Torreya grandis genome illuminates the origin and evolution of gymnosperm-specific sciadonic acid biosynthesis. Nat. Commun..

[9] Sheldon N.D., Smith S.Y., Stein R., Ng M. (2020). Carbon isotope ecology of gymnosperms and implications for paleoclimatic and paleoecological studies. Glob. Planet. Chang..

[10] Dror D., Klein T. (2022). The effect of elevated CO2 on aboveground and belowground carbon allocation and eco-physiology of four species of angiosperm and gymnosperm forest trees. Tree Physiol..

[11] Van Konijnenburg-van Cittert J.H., Pott C., Schmeißner S., Dütsch G., Kustatscher E. (2021). The Rhaetian flora of Wüstenwelsberg, Bavaria, Germany: Description of selected gymnosperms (Ginkgoales, Cycadales, Coniferales) together with an ecological assessment of the locally prevailing vegetation. Rev. Palaeobot. Palynol..

[12] Subedi S.C., Bhattarai K.R., Perez T.M., Sah J.P. (2020). Gymnosperm species richness patterns along the elevational gradient and its comparison with other plant taxonomic groups in the Himalayas. Front. Biogeogr..

[13] Nguyen T.T.T., Bae E.-K., Tran T.N.A., Lee H., Ko J.-H. (2023). Exploring the Seasonal Dynamics and Molecular Mechanism of Wood Formation in Gymnosperm Trees. Int. J. Mol. Sci..

[14] Swor K., Satyal P., Poudel A., Setzer W.N. (2023). Gymnosperms of Idaho: Chemical Compositions and Enantiomeric Distributions of Essential Oils of *Abies lasiocarpa*, *Picea engelmannii*, *Pinus contorta*, *Pseudotsuga menziesii*, and *Thuja plicata*. Molecules.

[15] Ng P.C., Kirkness E.F. (2010). Whole genome sequencing. Genetic Variation: Methods and Protocols.

[16] Maxam A.M., Gilbert W. (1977). A new method for sequencing DNA. Proc. Natl. Acad. Sci. USA.

[17] Behjati S., Tarpey P.S. (2013). What is next generation sequencing?. Arch. Dis. Child.-Educ. Pract..

[18] Schadt E.E., Turner S., Kasarskis A. (2010). A window into third-generation sequencing. Hum. Mol. Genet..

[19] McElhinney L.M., Marston D.A., Ellis R.J., Freuling C.M., Müller T.F., Fooks A.R. (2014). Sanger Sequencing of Lyssaviruses. Current Laboratory Techniques in Rabies Diagnosis, Research and Prevention.

[20] Crossley B.M., Bai J., Glaser A., Maes R., Porter E., Killian M.L., Clement T., Toohey-Kurth K. (2020). Guidelines for Sanger sequencing and molecular assay monitoring. J. Vet. Diagn. Investig..

[21] Metzker M.L. (2005). Emerging technologies in DNA sequencing. Genome Res..

[22] Blazej R.G., Kumaresan P., Mathies R.A. (2006). Microfabricated bioprocessor for integrated nanoliter-scale Sanger DNA sequencing. Proc. Natl. Acad. Sci. USA.

[23] Muzzey D., Kash S., Johnson J.I., Melroy L.M., Kaleta P., Pierce K.A., Ready K., Kang H.P., Haas K.R. (2019). Software-assisted manual review of clinical next-generation sequencing data: An alternative to routine Sanger sequencing confirmation with equivalent results in >15,000 germline DNA screens. J. Mol. Diagn..

[24] Menon S. (2021). Comparison of High-Throughput Next generation sequencing data processing pipelines. Int. Res. J. Mod. Eng. Technol. Sci. (IRJMETS).

[25] Saeed M., Jamil Z., Shehzad T., ul Hasan S.Z., Bibi R., Malik S.N., Matee-ur-Rehman H., Ahmed R. (2023). Role of Next Generation Sequencing (NGS) in Plant Disease Management: A Review. J. Appl. Res. Plant Sci..

[26] Cheng C., Fei Z., Xiao P. (2023). Methods to improve the accuracy of next-generation sequencing. Front. Bioeng. Biotechnol..

[27] Yang C.-Y., Yeh Y.-C., Wang L.-C., Lin Y.-Y., Lin S.-Y., Wang S.-Y., Chu P.-Y., Liu Z.-Y., Su Y.-C., Ho H.-L. (2023). Genomic Profiling With Large-Scale Next-Generation Sequencing Panels Distinguishes Separate Primary Lung Adenocarcinomas From Intrapulmonary Metastases. Mod. Pathol..

[28] Heydari A.A., Sindi S.S. (2023). Deep learning in spatial transcriptomics: Learning from the next next-generation sequencing. Biophys. Rev..

[29] Xu Z., Yan S., Yuan S., Wu C., Chen S., Guo Z., Li Y. (2023). Efficient Two-Stage Analysis for Complex Trait Association with Arbitrary Depth Sequencing Data. Stats.

[30] Hassan S., Bahar R., Johan M.F., Mohamed Hashim E.K., Abdullah W.Z., Esa E., Abdul Hamid F.S., Zulkafli Z. (2023). Next-generation sequencing (NGS) and third-generation sequencing (TGS) for the diagnosis of thalassemia. Diagnostics.

[31] Thun G.A., Gueuning M., Mattle-Greminger M. (2023). Long-read sequencing in blood group genetics. Transfus. Med. Hemother..

[32] Wang D., Cheng J., Peng L., Li Y. (2023). Comparison of Metagenomic Second-and Third-Generation Sequencing by Diagnostic Sensitivity and Specificity in Tuberculosis Patients. Clin. Lab..

[33] Zhuang J., Chen C., Fu W., Wang Y., Zhuang Q., Lu Y., Xie T., Xu R., Zeng S., Jiang Y. (2023). Third-Generation Sequencing as a New Comprehensive Technology for Identifying Rare α-and β-Globin Gene Variants in Thalassemia Alleles in the Chinese Population. Arch. Pathol. Lab. Med..

[34] Thudi M., Li Y., Jackson S.A., May G.D., Varshney R.K. (2012). Current state-of-art of sequencing technologies for plant genomics research. Brief. Funct. Genom..

[35] Athanasopoulou K., Boti M.A., Adamopoulos P.G., Skourou P.C., Scorilas A. (2021). Third-generation sequencing: The spearhead towards the radical transformation of modern genomics. Life.

[36] Wong L.L., Razali S.A., Deris Z.M., Danish-Daniel M., Tan M.P., Nor S.A.M., Ma H., Min W., Yantao L., Asaduzzaman M. (2022). Application of second-generation sequencing (SGS) and third generation sequencing (TGS) in aquaculture breeding program. Aquaculture.

[37] Saini M.K., Gaurav H.B., Kumar J., Sanu K. (2023). DNA Sequencing techniques: Sanger to Next Generation Sequencing. DNA.

[38] Tsiatis A.C., Norris-Kirby A., Rich R.G., Hafez M.J., Gocke C.D., Eshleman J.R., Murphy K.M. (2010). Comparison of Sanger sequencing, pyrosequencing, and melting curve analysis for the detection of KRAS mutations: Diagnostic and clinical implications. J. Mol. Diagn..

[39] Metzker M.L. (2010). Sequencing technologies—The next generation. Nat. Rev. Genet..

[40] Margulies M., Egholm M., Altman W.E., Attiya S., Bader J.S., Bemben L.A., Berka J., Braverman M.S., Chen Y.-J., Chen Z. (2005). Genome sequencing in microfabricated high-density picolitre reactors. Nature.

[41] Voelkerding K.V., Dames S.A., Durtschi J.D. (2009). Next-generation sequencing: From basic research to diagnostics. Clin. Chem..

[42] Luo C., Tsementzi D., Kyrpides N., Read T., Konstantinidis K.T. (2012). Direct comparisons of Illumina vs. Roche 454 sequencing technologies on the same microbial community DNA sample. PLoS ONE.

[43] Tawfik D.S., Griffiths A.D. (1998). Man-made cell-like compartments for molecular evolution. Nat. Biotechnol..

[44] Balasubramanian S. (2011). Sequencing nucleic acids: From chemistry to medicine. Chem. Commun..

[45] McKernan K.J., Peckham H.E., Costa G.L., McLaughlin S.F., Fu Y., Tsung E.F., Clouser C.R., Duncan C., Ichikawa J.K., Lee C.C. (2009). Sequence and structural variation in a human genome uncovered by short-read, massively parallel ligation sequencing using two-base encoding. Genome Res..

[46] Heather J.M., Chain B. (2016). The sequence of sequencers: The history of sequencing DNA. Genomics.

[47] Petersen L.M., Martin I.W., Moschetti W.E., Kershaw C.M., Tsongalis G.J. (2019). Third-generation sequencing in the clinical laboratory: Exploring the advantages and challenges of nanopore sequencing. J. Clin. Microbiol..

[48] Rhoads A., Au K.F. (2015). PacBio sequencing and its applications. Genom. Proteom. Bioinform..

[49] Ardui S., Ameur A., Vermeesch J.R., Hestand M.S. (2018). Single molecule real-time (SMRT) sequencing comes of age: Applications and utilities for medical diagnostics. Nucleic Acids Res..

[50] Zhao L., Zhang H., Kohnen M.V., Prasad K.V., Gu L., Reddy A.S. (2019). Analysis of transcriptome and epitranscriptome in plants using PacBio Iso-Seq and nanopore-based direct RNA sequencing. Front. Genet..

[51] Wang Y., Zhao Y., Bollas A., Wang Y., Au K.F. (2021). Nanopore sequencing technology, bioinformatics and applications. Nat. Biotechnol..

[52] Lu H., Giordano F., Ning Z. (2016). Oxford Nanopore MinION sequencing and genome assembly. Genom. Proteom. Bioinform..

[53] Magi A., Semeraro R., Mingrino A., Giusti B., D’aurizio R. (2018). Nanopore sequencing data analysis: State of the art, applications and challenges. Brief. Bioinform..

[54] Loman N.J., Quinlan A.R. (2014). Poretools: A toolkit for analyzing nanopore sequence data. Bioinformatics.

[55] Samarakoon H., Punchihewa S., Senanayake A., Hammond J.M., Stevanovski I., Ferguson J.M., Ragel R., Gamaarachchi H., Deveson I.W. (2020). Genopo: A nanopore sequencing analysis toolkit for portable Android devices. Commun. Biol..

[56] Ferguson J.M., Smith M.A. (2019). SquiggleKit: A toolkit for manipulating nanopore signal data. Bioinformatics.

[57] Pryszcz L.P., Novoa E.M. (2022). ModPhred: An integrative toolkit for the analysis and storage of nanopore sequencing DNA and RNA modification data. Bioinformatics.

[58] Tham C.-Y., Poon L., Yan T., Koh J.Y.P., Ramlee M.K., Teoh V.S.I., Zhang S., Cai Y., Hong Z., Lee G.S. (2023). High-throughput telomere length measurement at nucleotide resolution using the PacBio high fidelity sequencing platform. Nat. Commun..

[59] Hackl T., Hedrich R., Schultz J., Förster F. (2014). proovread: Large-scale high-accuracy PacBio correction through iterative short read consensus. Bioinformatics.

[60] Coupland P., Chandra T., Quail M., Reik W., Swerdlow H. (2012). Direct sequencing of small genomes on the Pacific Biosciences RS without library preparation. Biotechniques.

[61] Sikic M. (2023). Facilitating genome structural variation analysis. Nat. Methods.

[62] Kono N., Arakawa K. (2019). Nanopore sequencing: Review of potential applications in functional genomics. Dev. Growth Differ..

[63] Haque F., Li J., Wu H.-C., Liang X.-J., Guo P. (2013). Solid-state and biological nanopore for real-time sensing of single chemical and sequencing of DNA. Nano Today.

[64] Gu Z., Ying Y.-L., Cao C., He P., Long Y.-T. (2015). Accurate data process for nanopore analysis. Anal. Chem..

[65] Schlotter T., Kloter T., Nakatsuka N., Aramesh M., Voros J., Zambelli T. (2022). Interface nanopores as a flexible technology for next-generation single-molecule protein sensing. Biophys. J..

[66] Zhang H., Li H., Jain C., Cheng H., Au K.F., Li H., Aluru S. (2021). Real-time mapping of nanopore raw signals. Bioinformatics.

[67] Loose M., Malla S., Stout M. (2016). Real-time selective sequencing using nanopore technology. Nat. Methods.

[68] Nanopore O. (2012). Oxford Nanopore announcement sets sequencing sector abuzz. Nat. Biotechnol..

[69] Wick R.R., Judd L.M., Wyres K.L., Holt K.E. (2021). Recovery of small plasmid sequences via Oxford Nanopore sequencing. Microb. Genom..

[70] Yan L., Wang X., Liu H., Tian Y., Lian J., Yang R., Hao S., Wang X., Yang S., Li Q. (2015). The genome of Dendrobium officinale illuminates the biology of the important traditional Chinese orchid herb. Mol. Plant.

[71] Xu Z., Pu X., Gao R., Demurtas O.C., Fleck S.J., Richter M., He C., Ji A., Sun W., Kong J. (2020). Tandem gene duplications drive divergent evolution of caffeine and crocin biosynthetic pathways in plants. BMC Biol..

[72] Shang J., Tian J., Cheng H., Yan Q., Li L., Jamal A., Xu Z., Xiang L., Saski C.A., Jin S. (2020). The chromosome-level wintersweet (Chimonanthus praecox) genome provides insights into floral scent biosynthesis and flowering in winter. Genome Biol..

[73] Song B., Ning W., Wei D., Jiang M., Zhu K., Wang X., Edwards D., Odeny D.A., Cheng S. (2023). Plant genome resequencing and population genomics: Current status and future prospects. Mol. Plant.

[74] He J., Zhao X., Laroche A., Lu Z.-X., Liu H., Li Z. (2014). Genotyping-by-sequencing (GBS), an ultimate marker-assisted selection (MAS) tool to accelerate plant breeding. Front. Plant Sci..

[75] Huang X., Feng Q., Qian Q., Zhao Q., Wang L., Wang A., Guan J., Fan D., Weng Q., Huang T. (2009). High-throughput genotyping by whole-genome resequencing. Genome Res..

[76] Elshire R.J., Glaubitz J.C., Sun Q., Poland J.A., Kawamoto K., Buckler E.S., Mitchell S.E. (2011). A robust, simple genotyping-by-sequencing (GBS) approach for high diversity species. PLoS ONE.

[77] Chapman J.A., Mascher M., Buluç A., Barry K., Georganas E., Session A., Strnadova V., Jenkins J., Sehgal S., Oliker L. (2015). A whole-genome shotgun approach for assembling and anchoring the hexaploid bread wheat genome. Genome Biol..

[78] Takagi H., Tamiru M., Abe A., Yoshida K., Uemura A., Yaegashi H., Obara T., Oikawa K., Utsushi H., Kanzaki E. (2015). MutMap accelerates breeding of a salt-tolerant rice cultivar. Nat. Biotechnol..

[79] Wen H., Wang W., Jiang X., Wu M., Bai H., Wu C., Shen L. (2022). Transcriptome analysis to identify candidate genes related to chlorogenic acid biosynthesis during development of Korla fragrant pear in Xinjiang. Food Sci. Hum. Wellness.

[80] Yan H., Sun M., Zhang Z., Jin Y., Zhang A., Lin C., Wu B., He M., Xu B., Wang J. (2023). Pangenomic analysis identifies structural variation associated with heat tolerance in pearl millet. Nat. Genet..

[81] Tian T., Wang S., Yang S., Yang Z., Liu S., Wang Y., Gao H., Zhang S., Yang X., Jiang C. (2023). Genome assembly and genetic dissection of a prominent drought-resistant maize germplasm. Nat. Genet..

[82] Kumar S., Mohapatra T. (2021). Dynamics of DNA methylation and its functions in plant growth and development. Front. Plant Sci..

[83] Bai D., Yi C. (2019). Advances in the Profiling of Single-Cell DNA Modifications. Small Methods.

[84] Ni P., Huang N., Nie F., Zhang J., Zhang Z., Wu B., Bai L., Liu W., Xiao C.-L., Luo F. (2021). Genome-wide detection of cytosine methylations in plant from Nanopore data using deep learning. Nat. Commun..

[85] Alonso C., Perez R., Bazaga P., Medrano M., Herrera C.M. (2016). MSAP markers and global cytosine methylation in plants: A literature survey and comparative analysis for a wild-growing species. Mol. Ecol. Resour..

[86] Hu J., Cai J., Xu T., Kang H. (2022). Epitranscriptomic mRNA modifications governing plant stress responses: Underlying mechanism and potential application. Plant Biotechnol. J..

[87] Xu Y., Zhang M., Zhang Q., Yu X., Sun Z., He Y., Guo W. (2021). Role of main RNA methylation in hepatocellular carcinoma: N6-methyladenosine, 5-methylcytosine, and N1-methyladenosine. Front. Cell Dev. Biol..

[88] Zhang L., Rong W., Ma J., Li H., Tang X., Xu S., Wang L., Wan L., Zhu Q., Jiang B. (2022). Comprehensive analysis of DNA 5-methylcytosine and N6-adenine methylation by nanopore sequencing in hepatocellular carcinoma. Front. Cell Dev. Biol..

[89] Zhong Z.-D., Xie Y.-Y., Chen H.-X., Lan Y.-L., Liu X.-H., Ji J.-Y., Wu F., Jin L., Chen J., Mak D.W. (2023). Systematic comparison of tools used for m6A mapping from nanopore direct RNA sequencing. Nat. Commun..

[90] Wang Y., Wang H., Xi F., Wang H., Han X., Wei W., Zhang H., Zhang Q., Zheng Y., Zhu Q. (2020). Profiling of circular RNA N6-methyladenosine in moso bamboo (*Phyllostachys edulis*) using nanopore-based direct RNA sequencing. J. Integr. Plant Biol..

[91] Zhang Q., Liang Z., Cui X., Ji C., Li Y., Zhang P., Liu J., Riaz A., Yao P., Liu M. (2018). N6-methyladenine DNA methylation in Japonica and Indica rice genomes and its association with gene expression, plant development, and stress responses. Mol. Plant.

[92] Yang W., Meng J., Liu J., Ding B., Tan T., Wei Q., Yu Y. (2020). The N1-methyladenosine methylome of petunia mRNA. Plant Physiol..

[93] Zhang J., Sheng H., Hu C., Li F., Cai B., Ma Y., Wang Y., Ma Y. (2023). Effects of DNA methylation on gene expression and phenotypic traits in cattle: A review. Int. J. Mol. Sci..

[94] Liu S., Gao Y., Canela-Xandri O., Wang S., Yu Y., Cai W., Li B., Xiang R., Chamberlain A.J., Pairo-Castineira E. (2022). A multi-tissue atlas of regulatory variants in cattle. Nat. Genet..

[95] Zhou L., Ng H.K., Drautz-Moses D.I., Schuster S.C., Beck S., Kim C., Chambers J.C., Loh M. (2019). Systematic evaluation of library preparation methods and sequencing platforms for high-throughput whole genome bisulfite sequencing. Sci. Rep..

[96] Beck D., Ben Maamar M., Skinner M.K. (2022). Genome-wide CpG density and DNA methylation analysis method (MeDIP, RRBS, and WGBS) comparisons. Epigenetics.

[97] Li R., Hu F., Li B., Zhang Y., Chen M., Fan T., Wang T. (2020). Whole genome bisulfite sequencing methylome analysis of mulberry (Morus alba) reveals epigenome modifications in response to drought stress. Sci. Rep..

[98] Li J., Li C., Deng Y., Wei H., Lu S. (2023). Characteristics of Salvia miltiorrhiza methylome and the regulatory mechanism of DNA methylation in tanshinone biosynthesis. Hortic. Res..

[99] Zheng G., Hu S., Cheng S., Wang L., Kan L., Wang Z., Xu Q., Liu Z., Kang C. (2023). Factor of DNA methylation 1 affects woodland strawberry plant stature and organ size via DNA methylation. Plant Physiol..

[100] Shen Y., Zhang J., Liu Y., Liu S., Liu Z., Duan Z., Wang Z., Zhu B., Guo Y.-L., Tian Z. (2018). DNA methylation footprints during soybean domestication and improvement. Genome Biol..

[101] Maunakea A.K., Nagarajan R.P., Bilenky M., Ballinger T.J., D’Souza C., Fouse S.D., Johnson B.E., Hong C., Nielsen C., Zhao Y. (2010). Conserved role of intragenic DNA methylation in regulating alternative promoters. Nature.

[102] Serre D., Lee B.H., Ting A.H. (2010). MBD-isolated Genome Sequencing provides a high-throughput and comprehensive survey of DNA methylation in the human genome. Nucleic Acids Res..

[103] Rothkegel K., Sánchez E., Montes C., Greve M., Tapia S., Bravo S., Prieto H., Almeida A.M. (2017). DNA methylation and small interference RNAs participate in the regulation of MADS-box genes involved in dormancy in sweet cherry (*Prunus avium* L.). Tree Physiol..

[104] Dou L., Jia X., Wei H., Fan S., Wang H., Guo Y., Duan S., Pang C., Yu S. (2017). Global analysis of DNA methylation in young (J1) and senescent (J2) *Gossypium hirsutum* L. cotyledons by MeDIP-Seq. PLoS ONE.

[105] Paun O., Verhoeven K.J., Richards C.L. (2019). Opportunities and limitations of reduced representation bisulfite sequencing in plant ecological epigenomics. New Phytol..

[106] Robertson M., Richards C. (2015). Opportunities and challenges of next-generation sequencing applications in ecological epigenetics. Mol. Ecol..

[107] Yadav C.B., Pandey G., Muthamilarasan M., Prasad M. (2018). Epigenetics and epigenomics of plants. Plant Genet. Mol. Biol..

[108] Schmitz R.J., He Y., Valdés-López O., Khan S.M., Joshi T., Urich M.A., Nery J.R., Diers B., Xu D., Stacey G. (2013). Epigenome-wide inheritance of cytosine methylation variants in a recombinant inbred population. Genome Res..

[109] Smith Z.D., Meissner A. (2013). DNA methylation: Roles in mammalian development. Nat. Rev. Genet..

[110] Haghani A., Li C.Z., Robeck T.R., Zhang J., Lu A.T., Ablaeva J., Acosta-Rodríguez V.A., Adams D.M., Alagaili A.N., Almunia J. (2023). DNA methylation networks underlying mammalian traits. Science.

[111] Lu A.T., Fei Z., Haghani A., Robeck T.R., Zoller J.A., Li C.Z., Lowe R., Yan Q., Zhang J., Vu H. (2021). Universal DNA methylation age across mammalian tissues. Innov. Aging.

[112] Fang Q., Yuan Z., Hu H., Zhang W., Wang G., Wang X. (2023). Genome-wide discovery of circulating cell-free DNA methylation biomarkers for colorectal cancer detection. Clin. Epigenet..

[113] Klughammer J., Romanovskaia D., Nemc A., Posautz A., Seid C.A., Schuster L.C., Keinath M.C., Lugo Ramos J.S., Kosack L., Evankow A. (2023). Comparative analysis of genome-scale, base-resolution DNA methylation profiles across 580 animal species. Nat. Commun..

[114] Donoghue P.C., Harrison C.J., Paps J., Schneider H. (2021). The evolutionary emergence of land plants. Curr. Biol..

[115] Batalova A.Y., Putintseva Y.A., Sadovsky M.G., Krutovsky K.V. (2022). Comparative genomics of seasonal senescence in forest trees. Int. J. Mol. Sci..

[116] Haas M., Schreiber M., Mascher M. (2019). Domestication and crop evolution of wheat and barley: Genes, genomics, and future directions. J. Integr. Plant Biol..

[117] Song A., Su J., Wang H., Zhang Z., Zhang X., Van de Peer Y., Chen F., Fang W., Guan Z., Zhang F. (2023). Analyses of a chromosome-scale genome assembly reveal the origin and evolution of cultivated chrysanthemum. Nat. Commun..

[118] Shen Z., Li W., Li Y., Liu M., Cao H., Provart N., Ding X., Sun M., Tang Z., Yue C. (2021). The red flower wintersweet genome provides insights into the evolution of magnoliids and the molecular mechanism for tepal color development. Plant J..

[119] Dong Y., Duan S., Xia Q., Liang Z., Dong X., Margaryan K., Musayev M., Goryslavets S., Zdunić G., Bert P.-F. (2023). Dual domestications and origin of traits in grapevine evolution. Science.

[120] Ahmed H.I., Heuberger M., Schoen A., Koo D.-H., Quiroz-Chavez J., Adhikari L., Raupp J., Cauet S., Rodde N., Cravero C. (2023). Einkorn genomics sheds light on history of the oldest domesticated wheat. Nature.

[121] Li A., Yang Q., Li R., Dai X., Cai K., Lei Y., Jia K., Jiang Y., Zan L. (2023). Chromosome-level genome assembly for takin (Budorcas taxicolor) provides insights into its taxonomic status and genetic diversity. Mol. Ecol..

[122] Schneider H. (2023). Integrating genomics and conservation to safeguard plant diversity. Integr. Conserv..

[123] Barbour M.A., Kliebenstein D.J., Bascompte J. (2022). A keystone gene underlies the persistence of an experimental food web. Science.

[124] Hu Y., Yu Z., Gao X., Liu G., Zhang Y., Šmarda P., Guo Q. (2023). Genetic diversity, population structure, and genome-wide association analysis of ginkgo cultivars. Hortic. Res..

[125] Xiang Q.P., Tang J.Y., Yu J.G., Smith D.R., Zhu Y.M., Wang Y.R., Kang J.S., Yang J., Zhang X.C. (2022). The evolution of extremely diverged plastomes in *Selaginellaceae* (lycophyte) is driven by repeat patterns and the underlying DNA maintenance machinery. Plant J..

[126] Li L., Chen X., Fang D., Dong S., Guo X., Li N., Campos L., Chen X., Fang D., Dong S. (2022). Genomes shed light on the evolution of Begonia, a mega-diverse genus. New Phytol..

[127] Stansell Z., Björkman T. (2020). From landrace to modern hybrid broccoli: The genomic and morphological domestication syndrome within a diverse *B. oleracea* collection. Hortic. Res..

[128] Hung T.H., So T., Thammavong B., Chamchumroon V., Theilade I., Phourin C., Bouamanivong S., Hartvig I., Gaisberger H., Jalonen R. (2023). Range-wide differential adaptation and genomic vulnerability in critically endangered Asian rosewoods. Proc. Natl. Acad. Sci. USA.

[129] Sun Y., Deng T., Zhang A., Moore M.J., Landis J.B., Lin N., Zhang H., Zhang X., Huang J., Zhang X. (2020). Genome sequencing of the endangered *Kingdonia uniflora* (Circaeasteraceae, Ranunculales) reveals potential mechanisms of evolutionary specialization. IScience.

[130] Chen Y., Ma T., Zhang L., Kang M., Zhang Z., Zheng Z., Sun P., Shrestha N., Liu J., Yang Y. (2020). Genomic analyses of a “living fossil”: The endangered dove-tree. Mol. Ecol. Resour..

[131] Ma H., Liu Y., Liu D., Sun W., Liu X., Wan Y., Zhang X., Zhang R., Yun Q., Wang J. (2021). Chromosome-level genome assembly and population genetic analysis of a critically endangered rhododendron provide insights into its conservation. Plant J..

[132] Yang Y., Ma T., Wang Z., Lu Z., Li Y., Fu C., Chen X., Zhao M., Olson M.S., Liu J. (2018). Genomic effects of population collapse in a critically endangered ironwood tree Ostrya rehderiana. Nat. Commun..

[133] Liang Z., Myers Z.A., Petrella D., Engelhorn J., Hartwig T., Springer N.M. (2022). Mapping responsive genomic elements to heat stress in a maize diversity panel. Genome Biol..

[134] Moghaddam S.M., Oladzad A., Koh C., Ramsay L., Hart J.P., Mamidi S., Hoopes G., Sreedasyam A., Wiersma A., Zhao D. (2021). The tepary bean genome provides insight into evolution and domestication under heat stress. Nat. Commun..

[135] Mekonnen T., Haileselassie T., Tesfaye K. (2017). Identification, mapping and pyramiding of genes/quantitative trait loci (qtls) for durable resistance of crops to biotic stresses. J. Plant Pathol. Microbiol..

[136] Sallam A., Eltaher S., Alqudah A.M., Belamkar V., Baenziger P.S. (2022). Combined GWAS and QTL mapping revealed candidate genes and SNP network controlling recovery and tolerance traits associated with drought tolerance in seedling winter wheat. Genomics.

[137] Das G., Rao G.J., Varier M., Prakash A., Prasad D. (2018). Improved Tapaswini having four BB resistance genes pyramided with six genes/QTLs, resistance/tolerance to biotic and abiotic stresses in rice. Sci. Rep..

[138] Parihar A.K., Kumar J., Gupta D.S., Lamichaney A., Naik Sj S., Singh A.K., Dixit G.P., Gupta S., Toklu F. (2022). Genomics enabled breeding strategies for major biotic stresses in pea (*Pisum sativum* L.). Front. Plant Sci..

[139] Rao G.J., Reddy J.N., Variar M., Mahender A. (2016). Molecular breeding to improve plant resistance to abiotic stresses. Advances in Plant Breeding Strategies: Agronomic, Abiotic and Biotic Stress Traits.

[140] Laosatit K., Somta P., Chen X., Srinives P. (2020). Genomic approaches to biotic stresses. The Mungbean Genome.

[141] Liu P., Liu R., Xu Y., Zhang C., Niu Q., Lang Z. (2023). DNA cytosine methylation dynamics and functional roles in horticultural crops. Hortic. Res..

[142] Dowen R.H., Pelizzola M., Schmitz R.J., Lister R., Dowen J.M., Nery J.R., Dixon J.E., Ecker J.R. (2012). Widespread dynamic DNA methylation in response to biotic stress. Proc. Natl. Acad. Sci. USA.

[143] Cao S., Chen K., Lu K., Chen S., Zhang X., Shen C., Zhu S., Niu Y., Fan L., Chen Z.J. (2023). Asymmetric variation in DNA methylation during domestication and de-domestication of rice. Plant Cell.

[144] Cao Q., Huang L., Li J., Qu P., Tao P., Crabbe M.J.C., Zhang T., Qiao Q. (2022). Integrated transcriptome and methylome analyses reveal the molecular regulation of drought stress in wild strawberry (*Fragaria nilgerrensis*). BMC Plant Biol..

[145] Zhong L., Xu Y.-h., Wang J.-b. (2009). DNA-methylation changes induced by salt stress in wheat Triticum aestivum. Afr. J. Biotechnol..

[146] Shan X., Wang X., Yang G., Wu Y., Su S., Li S., Liu H., Yuan Y. (2013). Analysis of the DNA methylation of maize (*Zea mays* L.) in response to cold stress based on methylation-sensitive amplified polymorphisms. J. Plant Biol..

[147] Wan T., Liu Z., Leitch I.J., Xin H., Maggs-Kölling G., Gong Y., Li Z., Marais E., Liao Y., Dai C. (2021). The Welwitschia genome reveals a unique biology underpinning extreme longevity in deserts. Nat. Commun..

[148] Li J., Han F., Yuan T., Li W., Li Y., Wu H.X., Wei H., Niu S. (2023). The methylation landscape of giga-genome and the epigenetic timer of age in Chinese pine. Nat. Commun..

[149] Niu S., Li J., Bo W., Yang W., Zuccolo A., Giacomello S., Chen X., Han F., Yang J., Song Y. (2022). The Chinese pine genome and methylome unveil key features of conifer evolution. Cell.

[150] Gao C., Deng M., Yang X., Yu W., Cai J., Shi Y., Zhu Z., Zhou T., Xue L., Cao F. (2020). Genome-Wide Identification and Coexpression Network Analysis of DNA Methylation Pathway Genes and Their Differentiated Functions in *Ginkgo biloba* L. Forests.

[151] Castander-Olarieta A., Pereira C., Sales E., Meijón M., Arrillaga I., Cañal M.J., Goicoa T., Ugarte M.D., Moncaleán P., Montalbán I.A. (2020). Induction of radiata pine somatic embryogenesis at high temperatures provokes a long-term decrease in DNA methylation/hydroxymethylation and differential expression of stress-related genes. Plants.

[152] Zhou X., Liu Z. (2022). Unlocking plant metabolic diversity: A (pan)-genomic view. Plant Commun..

[153] Shang X., Yi X., Xiao L., Zhang Y., Huang D., Xia Z., Ou K., Ming R., Zeng W., Wu D. (2022). Chromosomal-level genome and multi-omics dataset of *Pueraria lobata* var. thomsonii provide new insights into legume family and the isoflavone and puerarin biosynthesis pathways. Hortic. Res..

[154] Zhu M., Wang Z., Yang Y., Wang Z., Mu W., Liu J. (2023). Multi-omics reveal differentiation and maintenance of dimorphic flowers in an alpine plant on the Qinghai-Tibet Plateau. Mol. Ecol..

[155] Li C., Wood J.C., Vu A.H., Hamilton J.P., Rodriguez Lopez C.E., Payne R.M., Serna Guerrero D.A., Gase K., Yamamoto K., Vaillancourt B. (2023). Single-cell multi-omics in the medicinal plant Catharanthus roseus. Nat. Chem. Biol..

[156] Neale D.B., Langley C.H., Salzberg S.L., Wegrzyn J.L. (2013). Open access to tree genomes: The path to a better forest. Genome Biol..

[157] Birol I., Raymond A., Jackman S.D., Pleasance S., Coope R., Taylor G.A., Yuen M.M.S., Keeling C.I., Brand D., Vandervalk B.P. (2013). Assembling the 20 Gb white spruce (*Picea glauca*) genome from whole-genome shotgun sequencing data. Bioinformatics.

[158] Stevens K.A., Wegrzyn J.L., Zimin A., Puiu D., Crepeau M., Cardeno C., Paul R., Gonzalez-Ibeas D., Koriabine M., Holtz-Morris A.E. (2016). Sequence of the sugar pine megagenome. Genetics.

[159] Neale D.B., McGuire P.E., Wheeler N.C., Stevens K.A., Crepeau M.W., Cardeno C., Zimin A.V., Puiu D., Pertea G.M., Sezen U.U. (2017). The Douglas-fir genome sequence reveals specialization of the photosynthetic apparatus in Pinaceae. G3: Genes Genomes Genet..

[160] Mosca E., Cruz F., Gómez-Garrido J., Bianco L., Rellstab C., Brodbeck S., Csilléry K., Fady B., Fladung M., Fussi B. (2019). A reference genome sequence for the European silver fir (*Abies alba* Mill.): A community-generated genomic resource. G3 Genes Genomes Genet..

[161] Kuzmin D.A., Feranchuk S.I., Sharov V.V., Cybin A.N., Makolov S.V., Putintseva Y.A., Oreshkova N.V., Krutovsky K.V. (2019). Stepwise large genome assembly approach: A case of Siberian larch (*Larix sibirica* Ledeb). BMC Bioinform..

[162] Cheng J., Wang X., Liu X., Zhu X., Li Z., Chu H., Wang Q., Lou Q., Cai B., Yang Y. (2021). Chromosome-level genome of Himalayan yew provides insights into the origin and evolution of the paclitaxel biosynthetic pathway. Mol. Plant.

[163] Xiong X., Gou J., Liao Q., Li Y., Zhou Q., Bi G., Li C., Du R., Wang X., Sun T. (2021). The Taxu s genome provides insights into paclitaxel biosynthesis. Nat. Plants.

[164] Song C., Fu F., Yang L., Niu Y., Tian Z., He X., Yang X., Chen J., Sun W., Wan T. (2021). Taxus yunnanensis genome offers insights into gymnosperm phylogeny and taxol production. Commun. Biol..

[165] Scott A.D., Zimin A.V., Puiu D., Workman R., Britton M., Zaman S., Caballero M., Read A.C., Bogdanove A.J., Burns E. (2020). A reference genome sequence for giant sequoia. G3 Genes Genomes Genet..

[166] Nystedt B., Street N.R., Wetterbom A., Zuccolo A., Lin Y.-C., Scofield D.G., Vezzi F., Delhomme N., Giacomello S., Alexeyenko A. (2013). The Norway spruce genome sequence and conifer genome evolution. Nature.

[167] Sun C., Xie Y.H., Li Z., Liu Y.J., Sun X.M., Li J.J., Quan W.P., Zeng Q.Y., Van de Peer Y., Zhang S.G. (2022). The Larix kaempferi genome reveals new insights into wood properties. J. Integr. Plant Biol..

[168] Liu H., Wang X., Wang G., Cui P., Wu S., Ai C., Hu N., Li A., He B., Shao X. (2021). The nearly complete genome of Ginkgo biloba illuminates gymnosperm evolution. Nat. Plants.

[169] Wan T., Liu Z.-M., Li L.-F., Leitch A.R., Leitch I.J., Lohaus R., Liu Z.-J., Xin H.-P., Gong Y.-B., Liu Y. (2018). A genome for gnetophytes and early evolution of seed plants. Nat. Plants.

[170] Liu Y., Wang S., Li L., Yang T., Dong S., Wei T., Wu S., Liu Y., Gong Y., Feng X. (2022). The Cycas genome and the early evolution of seed plants. Nat. Plants.

[171] Fu F., Song C., Wen C., Yang L., Guo Y., Yang X., Shu Z., Li X., Feng Y., Liu B. (2023). The Metasequoia genome and evolutionary relationships among redwoods. Plant Commun..

[172] Scott A.D., Stenz N.W., Ingvarsson P.K., Baum D.A. (2016). Whole genome duplication in coast redwood (*Sequoia sempervirens*) and its implications for explaining the rarity of polyploidy in conifers. New Phytol..

[173] Yue M., Chen H., Xuan L., Yang Y., Chong X., Li M., Yu C., Lu X., Zhang F. (2023). Novel molecular markers for Taxodium breeding from the chloroplast genomes of four artificial Taxodium hybrids. Front. Genet..

[174] Chen L., Li L., Yang G., Qian H., Li M. (2019). Characterization of the complete chloroplast genome sequence of *Tsuga longibracteata* WC Cheng (Pinaceae). Conserv. Genet. Resour..

[175] Wang Y., Wang Z., Zhang L., Yang X. (2018). The complete chloroplast genome of *Cycas Szechuanensis*, an extremely endangered species. Mitochondrial DNA.

[176] Yang X., Deng T., Tang W., Wu T. (2022). Characterization of the complete chloroplast genome of *Cycas ferruginea*, a vulnerable species. Mitochondrial DNA.

[177] Lin C.-P., Wu C.-S., Huang Y.-Y., Chaw S.-M. (2012). The complete chloroplast genome of *Ginkgo biloba* reveals the mechanism of inverted repeat contraction. Genome Biol. Evol..

[178] Li J. (2019). The complete chloroplast genome sequence of *Podocarpus imbricatus* (Podocarpaceae) and its phylogenetic analysis. Mitochondrial DNA.

[179] Li J., Xu B., Yang Q., Liu Z.-L. (2020). The complete chloroplast genome sequence of *Picea schrenkiana* (Pinaceae). Mitochondrial DNA.

[180] Parmar R., Cattonaro F., Phillips C., Vassiliev S., Morgante M., Rajora O.P. (2022). Assembly and annotation of Red Spruce (*Picea rubens*) chloroplast genome, identification of simple sequence repeats, and phylogenetic analysis in Picea. Int. J. Mol. Sci..

[181] Yang M.-Q., Du Y., Ling L.-Z. (2018). Characterization of the complete chloroplast genome of *Pinus wangii* (Pinaceae), an endangered and endemic species in China. Mitochondrial DNA.

[182] Wu L.-X., Wang Y.-H., Gong X. (2021). Characterization of the complete chloroplast genome of *Cycas hongheensis* (Cycadaceae), an endemic species in the red river region of China. Mitochondrial DNA.

[183] Kersten B., Rellstab C., Schroeder H., Brodbeck S., Fladung M., Krutovsky K.V., Gugerli F. (2022). The mitochondrial genome sequence of *Abies alba* Mill. reveals a high structural and combinatorial variation. BMC Genom..

[184] Li J., Milne R.I., Ru D., Miao J., Tao W., Zhang L., Xu J., Liu J., Mao K. (2020). Allopatric divergence and hybridization within *Cupressus chengiana* (Cupressaceae), a threatened conifer in the northern Hengduan Mountains of western China. Mol. Ecol..

[185] WANG H.W., Ge S. (2006). Phylogeography of the endangered *Cathaya argyrophylla* (Pinaceae) inferred from sequence variation of mitochondrial and nuclear DNA. Mol. Ecol..

[186] Meena B., Singh N., Mahar K.S., Sharma Y.K., Rana T.S. (2019). Molecular analysis of genetic diversity and population genetic structure in *Ephedra foliata*: An endemic and threatened plant species of arid and semi-arid regions of India. Physiol. Mol. Biol. Plants.

[187] Sanger F., Nicklen S., Coulson A.R. (1977). DNA sequencing with chain-terminating inhibitors. Proc. Natl. Acad. Sci. USA.

[188] Guo W., Cannon A., Lisch D. (2022). A Molecular Cloning and Sanger Sequencing-based Protocol for Detecting Site-specific DNA Methylation. Bio-Protocol.

[189] Park J.-S., Kang M.-Y., Shim E.-J., Oh J., Seo K.-I., Kim K.S., Sim S.-C., Chung S.-M., Park Y., Lee G.P. (2022). Genome-wide core sets of SNP markers and Fluidigm assays for rapid and effective genotypic identification of Korean cultivars of lettuce (*Lactuca sativa* L.). Hortic. Res..

[190] Wang S., Wang K., Li Z., Li Y., He J., Li H., Wang B., Xin T., Tian H., Tian J. (2022). Architecture design of cucurbit crops for enhanced productivity by a natural allele. Nat. Plants.

[191] Gao L., Xu W., Xin T., Song J. (2023). Application of third-generation sequencing to herbal genomics. Front. Plant Sci..

[192] Shen L., Ding C., Zhang W., Zhang T., Li Z., Zhang J., Chu Y., Su X. (2023). The Populus koreana genome provides insights into the biosynthesis of plant aroma. Ind. Crops Prod..

[193] Xu D., Zhang J., Zhao X., Jiang H., Ma X., Pan W. (2022). CIDP: A multi-functional platform for designing CRISPR sgRNAs. Hortic. Res..

[194] Xu D., Jin K., Jiang H., Gong D., Yang J., Yu W., Yang Y., Li J., Pan W. (2023). GFAP: Ultra-fast and accurate gene functional annotation software for plants. Plant Physiol..

[195] Sun Y., Shang L., Zhu Q.-H., Fan L., Guo L. (2022). Twenty years of plant genome sequencing: Achievements and challenges. Trends Plant Sci..

